# The loss-of-function of DNA methyltransferase 1 by siRNA impairs the growth of non-small cell lung cancer with alleviated side effects via reactivation of RASSF1A and APC *in vitro* and *vivo*

**DOI:** 10.18632/oncotarget.19573

**Published:** 2017-07-26

**Authors:** Qi Lai, Yin-Hui Xu, Qiang Chen, Liang Tang, An-Gui Li, Li-Fei Zhang, Chun-Fang Zhang, Jian-Fei Song, Zhen-Zong Du

**Affiliations:** ^1^ Department of Thoracic Surgery, Xiangya Hospital of Central South University, Changsha 410008, P.R. China; ^2^ Department of Thoracic Surgery, The Second Affiliated Hospital of Guilin Medical University, Guilin 541199, P.R. China; ^3^ Department of Thoracic Surgery, The Affiliated Hospital of Guilin Medical University, Guilin 541001, P.R. China; ^4^ Department of Thoracic Surgery, Nanxi Shan Hospital of Guangxi Zhuang Autonomous Region, The Affiliated Nanxi Shan Hospital of Guilin Medical University, Guilin 541002, P.R. China

**Keywords:** DNMT1, 5-Aza-CR, siRNA, lung cancer, side effects

## Abstract

Hypermethylation of tumor suppressor genes (TSGs) promoters by DNA methyltransferase (DNMT) can be observed in almost all cancers which represent a hallmark of carcinogenesis, including lung cancer. DNMT inhibitors (e.g.5-Aza-CR/CdR) reactivate TSGs to exert anti-cancer activity and have been applied into the clinical. However, it is cytotoxic even at low concentrations, which might be not directly related to DNA methylation. We here investigated an alternative strategy in the lung cancer therapy and aimed to estimate and compare its efficiency and side effects of knockdown of DNMT1 *in vitro* and *in vivo*. Lung cancer tissues (n=20) showed enhanced expression of DNMT1 than corresponding non-neoplastic tissues. Similar results were found in lung cancer cell lines A549 and H538. The treatment of 5-Aza-CR or knockdown of DNMT1 *in vitro* could inhibit the expressions of DNMT1 but restore the TSGs expressions including the Ras association domain family 1A (RASSF1A) and the adenomatous polyposis coli (APC) via the demethylation of its promoter region, which results in the decreased proliferation, increased apoptosis and impaired ability of migration. Importantly, knockdown of DNMT1 by siRNA *in vivo* also effectively demethylated the RASSF1A and APC promoter, elevated their expressions and limited tumor growth, which functioned like 5-Aza-CR but with alleviated side effects, suggesting that knockdown of DNMT1 might be potential strategy for the treatment of lung cancer with better tolerability.

## INTRODUCTION

As a global health burden, lung cancer remains the leading cause of cancer-related deaths worldwide with 221,200 estimated new cases, and accounting for 1.59 million deaths annually [1]. Clinically, lung cancer is classically divided into two major histological subtypes including non-small-cell lung cancer (NSCLC) and small-cell lung cancer (SCLC), with the former accounting for approximately 80% of the cases and the remaining 20% of cases are SCLC. The diagnosis usually made at an advanced stage is the main contributor to the low survival rate of lung cancer with limited therapeutic options [2, 3]. The platinum-based chemotherapy and radiotherapy are regular treatments for patients with lung cancer, and the targeted anticancer therapy and immune checkpoint inhibitors have also been pay many attentions in recent decades. However, the costs of immunotherapy are far more than conventional therapy and only 15-25% of NSCLC patients derive benefit from immunotherapy [4]. Therefore, novel strategy for the treatment of lung cancer should be considered and investigated.

Epigenetic disorders have been demonstrated to correlate with many kinds of significant human diseases including carcinogenesis, neuron disorder and cardiovascular diseases [5–7]. Aberrant DNA methylation patterns via DNA methyltransferases (DNMTs) are frequently found in human malignancies and result in epigenetic silencing of tumor suppressor genes (TSGs) by transcriptional silencing. Five types of DNMTs have been identified in mammalian genomes, viz. DNMT1, 2, 3A, 3B, and 3L [8]. Mounting evidences indicated that abnormal expression of DNMTs could aberrantly hyper-methylate and inhibit of TSGs, leading to promoting the progression of lung cancer [9]. Indeed, approximately 400 methylation-silenced genes in a single tumor have been found, such as the Ras association domain family 1A (RASSF1A), CHD13 and the adenomatous polyposis coli (APC) [10]. RASSF1A and APC were reported to regulate the carcinogenesis via cell cycle control, microtubule stabilization, cellular adhesion and mortality, as well as apoptosis [11, 12]. Therefore, abrogation the hypermethylation of TSGs to restore its tumor suppressive ability could be the effective ways for lung cancer treatments.

DNMT inhibitors are the class of drugs that inhibit the enzyme DNA methyltransferase and were first discovered to be cytotoxic agents, but with poor efficacy and tolerability [13, 14]. Subsequently, its demethylating activity has been demonstrated to restore normal growth and differentiation by demethylation of tumor suppressor genes [15]. The two best studied DNMT inhibitors are azacytidine (5-Azacytidine; 5-Aza-CR) and deoxycytidine (5-Aza-2’-deoxycytidine; 5-Aza-CdR) and have been applied into the clinical [16].

Among the novel agents of demethylation, the most intensively studied are DNMTs antisense and siRNA. However, the efficiency of siRNA-DNMTs needs more investigations. In hepatocellular carcinoma (HCC), Fan et al. has reported that knock-down of DNMT1 expression by siRNA induced the promoter of the tumor suppressor gene CDH1 demethylation and upregulated CDH1 transcription [17]. In addition, Jung et al. found that DNMT1-targeted inhibition by siRNA induced the re-expression and reversed DNA methylation including RASSF1A etc., and could also inhibit cell proliferation in the cancer cells *in vitro* with no DNA damage, but 5-Aza-CdR showed more cytotoxicity than siRNA-DNMT1 with significant DNA damage, which was harm to the normal cells [18]. Furthermore, the investigations *in vivo* on the efficiency of siRNA-DNMT1 were needed, especially in the lung cancer.

In present study, we found up-regulated expression of DNMT1 in lung cancer tissues and cell lines. Results *in vitro* showed that knockdown of DNMT1 by siRNA, similar to the 5-Aza-CR, could re-express RASSF1A and APC by demethylating their promoter regions, and could promote apoptosis, inhibit proliferation and migration of two lung cancer cell lines. Moreover, we demonstrated that knockdown of DNMT1 *in vivo* could also enhance the RASSF1A and APC expression via demethylation, and delayed the lung cancer growth with reduced adverse side effects. These findings implicated that siRNA-DNMT1 could be a potential treatment for lung cancer therapy.

## RESULTS

### 5-Aza-CR inhibits DNMT1 to re-express RASSF1A and APC via demethylation in lung cancer cell lines

**Figure 1:**
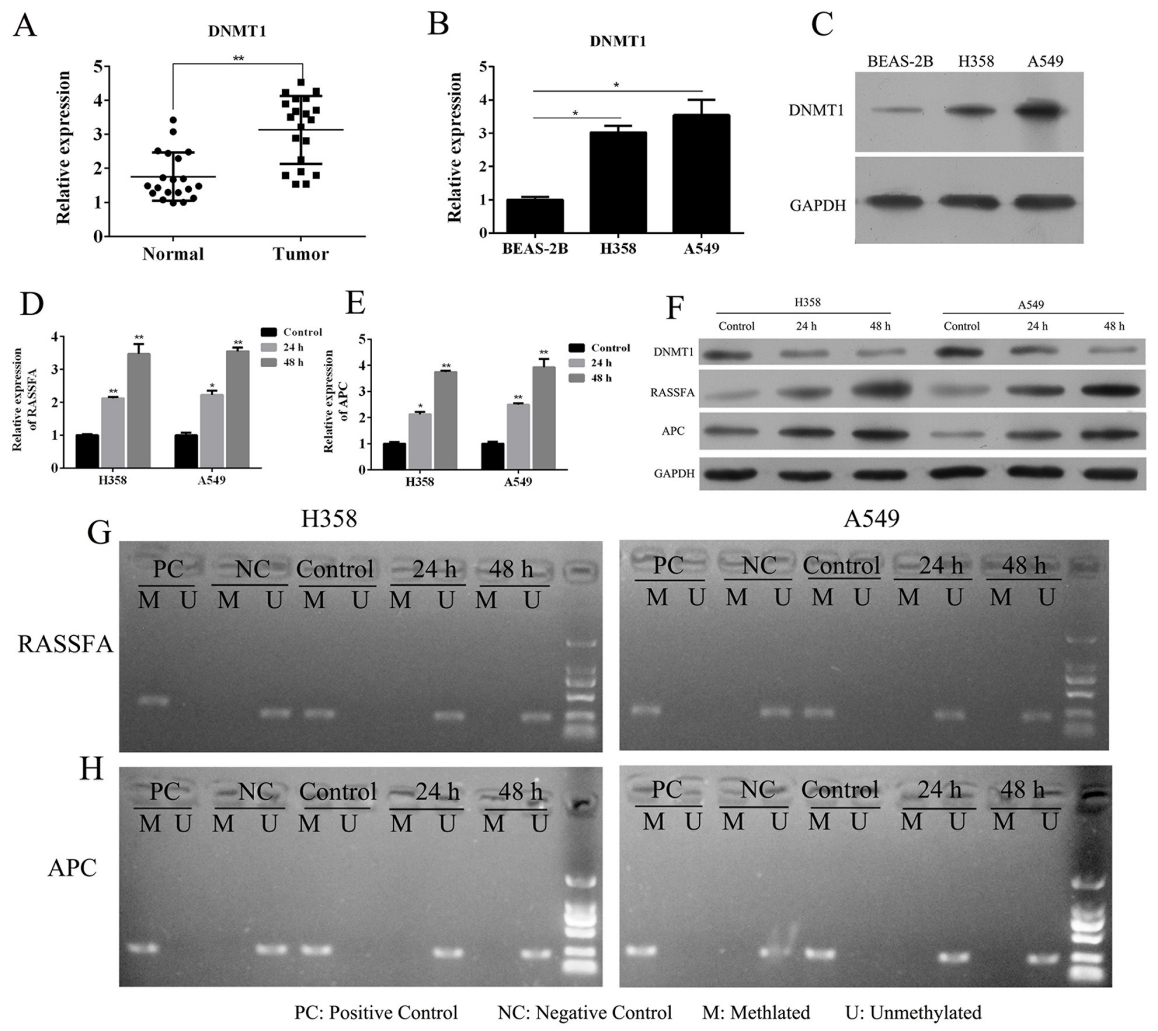
The mRNA **(A)** expression of DNMT1 in lung cancer tissues and corresponding paracancerous tissues were determined by Q-PCR. The mRNA **(B)** and protein **(C)** expression of DNMT1 in lung cancer cell lines A549 and H358 or normal lung epithelial cells BEAS-2B were determined by Q-PCR and WB. A549 and H358 cells were treated with 3 μM 5-Aza-CR and then the mRNA expression **(D-E)** and protein expression of RASSF1A and APC **(F)**, and its methylation status **(G-H)** in lung cancer cell lines A549 and H358 or normal lung epithelial cells BEAS-2B were determined by MSP, Q-PCR and WB.*P< 0.05, **P< 0.01, ***P< 0.001, data represent the means ± s.d.

To investigate the loss-of-function of DNMT1 in lung cancer, the DNMTs inhibitor 5-Aza-CR was used. We here collected lung cancer tissues (n=20) and corresponding non-neoplastic tissues (n=20) for the Q-PCR analysis. The results showed that the expression of DNMT1 was significantly up-regulated in NSCLC than that in the corresponding non-neoplastic tissue (Figure 1A). The expression of DNMT1 was also confirmed in normal lung epithelial cells and two lung cancer cell lines, A549 and H358 has higher expression of DNMT1 than BEAS-2B in mRNA and protein level (Figure 1B and 1C). The treatment of 5-Aza-CR in A549 and H358 for 24, 48h could significantly impair the DNMT1 expression and demethylated the promoter regions of RASSF1A and APC (Figure 1D and 1E), which leading to up-regulate the RASSF1A and APC expression in a time-dependent manner by demethylating RASSF1A and APC (Figure 1F-1H). These data indicated that the DNMTs inhibitor 5-Aza-CR could restore the tumor suppressor genes RASSF1A and APC expression by inhibit DNMT1.

### The treatment of 5-Aza-CR inhibits proliferation, migration and induces apoptosis of lung cancer cells

**Figure 2:**
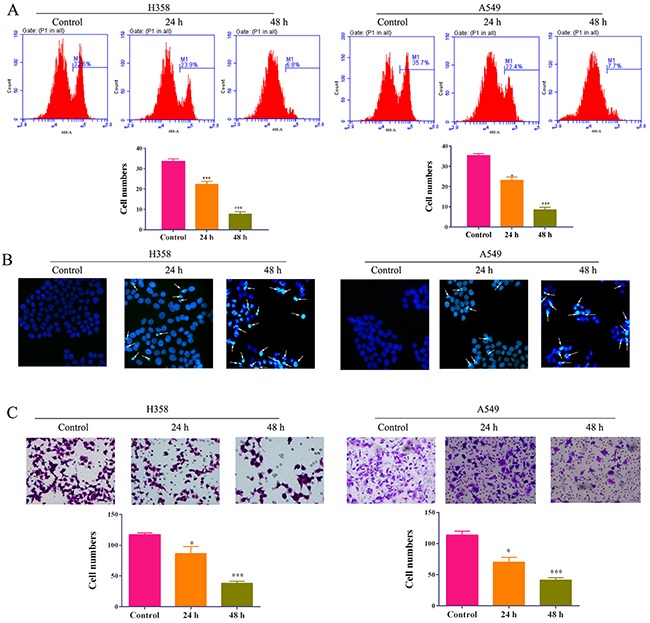
The proliferation **(A)**, apoptosis **(B)** and migration **(C)** of lung cancer cell lines A549 and H358 cells treated with 5-Aza-CR were determined by BrdU assay, hochestassay and transwell assay, respectively.

As the important tumor suppressor genes, RASSF1A and APC were found to involve into cellular adhesion, apoptosis and cell cycle arrest. Therefore, we estimated the impacts of 5-Aza-CR on A549 and H358 cells including proliferation, apoptosis and migration. BrdU proliferation assay indicated that the proliferation of A549 and H358 was remarkably reduced by 5-Aza-CR for 24h and 48h (Figure 2A). Additionally, the hochest assay showed that the addition of 5-Aza-CR could promote the cell apoptosis (Figure 2B). Furthermore, the ability of migration was assessed by transwell assay which demonstrated that 5-Aza-CR could also decrease the capability of migration of two lung cancer cell lines (Figure 2C). The results suggested that 5-Aza-CR is an effective agent for suppressing tumor growth and migration of lung cancer.

### Knockdown of DNMT1 also negatively regulates RASSF1A and APC via demethylation

**Figure 3:**
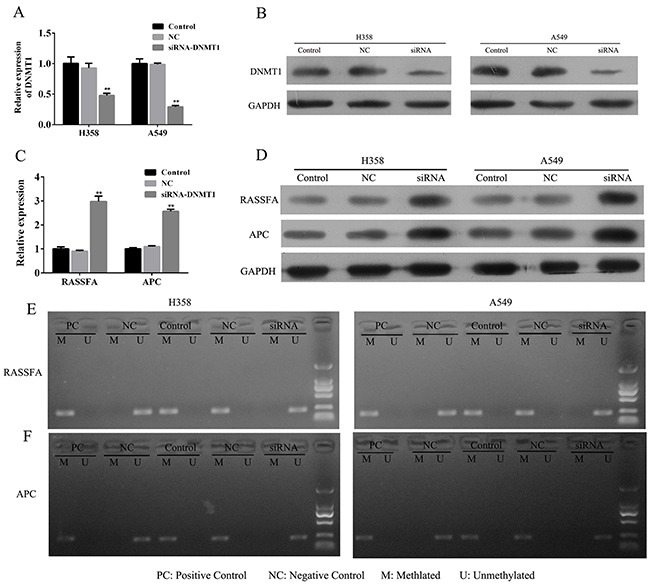
The lung cancer cells A549 and H358 were transfected with siRNA-DNMT1 or negative control and the mRNA **(A)** and protein expression **(B)** of DNMT1 and RASSF1A and APC **(C-D)** were assessed by Q-PCR and WB. The status of methylationof RASSF1A and APC were determined by MSP **(E-F)**. **P< 0.01, data represent the means ± s.d.

To further clarify the efficiency of loss-of-function of DNMT1, we transfected the A549 and H358 cells with siRNA for DNMT1 for 48h to successively knockdown of DNMT1 (Figure 3A and 3B). The results from Q-PCR showed that knockdown of DNMT1 for 48 h significantly promoted the mRNA expression of RASSF1A and APC (Figure 3C). Meanwhile, Weston blot also confirmed that the protein level of RASSF1A and APC were up-regulated by the siRNA-DNMT1 (Figure 3D). To test the possibility that siRNA-DNMT1 demethylated the promoters of RASSF1A and APC, we estimated the status of methylation of two lung cancer cells and found that RASSF1A and APC were demethylated by DNMT1 knockdown for 48h (Figure 3E and 3F), which suggested that loss-of-function of DNMT1 by siRNA could effectively reactivate the tumor suppressive genes RASSF1A and APC via demethylation.

### Loss-of-function of DNMT1 by siRNA could limit the growth of lung cancer cells

**Figure 4:**
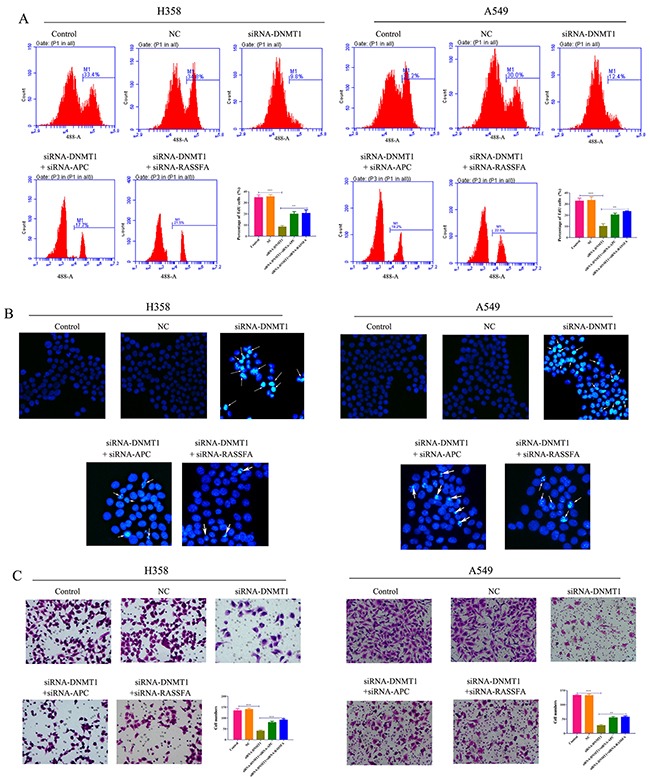
The proliferation **(A)**, apoptosis **(B)** and migration **(C)** of lung cancer cell lines A549 and H358 cells transfected with siRNA-DNMT1 and/or siRNA-APC/RASSF1A, negative control were determined by BrdU assay, hochest assay and transwell assay, respectively.

We have demonstrated that the inhibitor of DNMT1 significantly promoted the apoptosis and inhibited the proliferation and migration ofA549 and H358 cells. Therefore, we investigated the impacts of siRNA-DNMT1 on lung cancer cells and found that the siRNA-DNMT1 showed the similar effects as 5-Aza-CR to constrict the growth and migration of tumor cells. BrdU proliferation assay indicated that the proliferation of A549 and H358 was remarkably reduced by siRNA-DNMT1 for 48h (Figure 4A). However, this effect of siRNA-DNMT1 was found to be mediated by APC and RASSF1A. The hochest assay showed that transfection of siRNA-DNMT1 could promote the cell apoptosis (Figure 4B). Furthermore, the ability of migration was assessed by transwell assay which demonstrated that siRNA-DNMT1 could also impair the capability of migration of two lung cancer cell lines via APC and RASSF1A (Figure 4C). The results *in vitro* implicated that, similar to the DNMT1 inhibitor 5-Aza-CR, loss-of-function of DNMT1 by siRNA was able to inhibit the growth and migration of lung cancer cells.

### siRNA-DNMT1 *in vivo* delay the progression of lung cancer with reduced side effects

**Figure 5:**
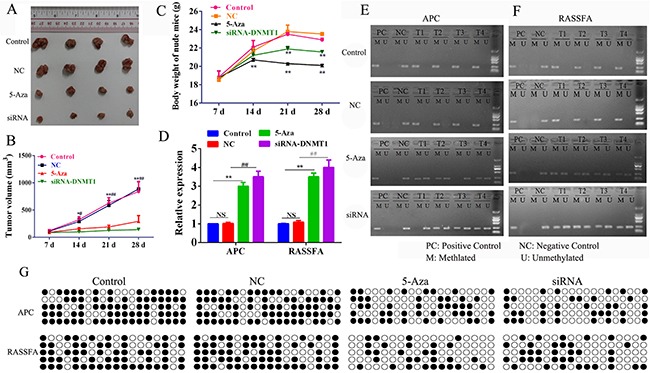
Nude mice (6 per group) were inoculated subcutaneously with 3×106 A549 cells and treated i.p. 500 nM /dose 5-Aza-CR or 10 nM siRNA-DNMT1 or negative control The tumor volume (mm^3^) **(A-B)** and body weight **(C)** were measured. After sacrifice, the tumor tissues were collected to analyze the expression **(D)** and the status of methylation of RASSF1A and APC **(E-G)**. *P< 0.05, **P< 0.01, ***P< 0.001. Data represent the means ± s.d.

We next assessed and compared the efficiency and adverse side effects of 5-Aza-CR and siRNA-DNMT1 *in vivo*. The xenograft model of human A549 was established and the nude mice were treated with negative control, 5-Aza-CR or siRNA-DNMT1. The results showed that the tumor volume was decreased comparably between the 5-Aza-CR and siRNA-DNMT1 groups (Figure 5A and 5B). We also estimated the body weight of each group and found that the mice treated with siRNA-DNMT1 groups were heavier than the mice treated with 5-Aza-CR (Figure 5C), which suggested that the siRNA-DNMT1 was more tolerable than the DNMT1 inhibitor 5-Aza-CR. Furthermore, we also determined the status of methylation (Figure 5D) and expression of RASSF1A and APC. The tumor tissues from each group were collected and the Q-PCR results showed that all the siRNA-DNMT1 and 5-Aza-CR enhanced the expression of RASSF1A and APC by demethylation (Figure 5E-5G).

## DISCUSSION

In China, lung cancer has being the most common incident cancer and the leading cause of cancer death in 2015 [19]. Retrospective data showed that the prognosis for advanced-stage lung cancer is very poor, with a 5-year overall survival rate of 17%. Besides, for widely disseminated disease, the 5-year overall survival rate only has 4.2% [20]. The conventional treatment of NSCLC has been cytotoxic chemotherapy which is gradually changed by the development of immunotherapy agents, e.g. anti-PD-1/L1 antibody, nivolumab and pembrolizumab [21]. However, the cost of immunotherapy is considered as a burden for tumor patients. Thus, the discovery of new ways or targets is necessary for patients to adopt appropriate cancer-therapy. In this study, we focused on the regulation of DNA methylation and compared the efficiency and side effects between DNMT1 inhibitor 5-Aza-CR and the siRNA-DNMT1for investigating pre-clinical applicability of the latter strategy. We found that 5-Aza-CR exerts its function by demethylation and other pathway which was related to significant cytotoxicity and poor tolerability, and the treatment of siRNA-DNMT1 could be the alternative lung cancer therapy.

Epimutations referred to the hypermethylation and epigenetic silencing of tumor suppressor genes [22]. DNA methylation as the first type of epigenetic mark is the most characterized epigenetic phenomenon that takes place almost exclusively at the carbon-5 position of cytosine residues within CpG dinucleotides and is carried out by DNA methyltransferases (DNMTs) with S-adenosyl-methionine (SAM) as the methyl donor [23]. DNA methyltransferase (DNMT) is the key enzyme involved in hypermethylation, including DNMT1, 2, 3A, 3B, and 3L, which were repored to be dysregulated in oral cancer [24], ovarian cancer [25], breast carcinoma [26] and lung cancer [27]. DNMT1 preferentially is responsible for maintenance of an established DNA methylation pattern and methylates newly biosynthesized DNA, while the DNMT3A and DNMT3b possess an efficient de novo methylation activity. We here also confirmed that the expression of DNMT1 is up-regulated in tumor tissues from NSCLC, and lung cancer cell lines.

Despite an overall reduction of DNA methylation (global hypomethylation), hypermethylation of CpG islands of tumor suppressor gene promoters was found in almost all cancers, which lead to transcriptional silencing and result in tumorigenesis, such as those involved in cell cycle regulation (APC, p16 INK4a, p14ARF), DNA repair (BRCA1, MGMT), apoptosis (RASSF1A, DAPK, TMS1) [1]. Therefore, inhibition of hypemethylation of TSGs is conducive to restore its tumor suppressive ability. DNMT inhibitors azacytidine (5-Azacytidine; 5-Aza-CR) and deoxycytidine (5-Aza-2’-deoxycytidine; 5-Aza-CdR) were found to inhibit DNA methyltransferase to restore normal growth and differentiation by demethylation of TSGs, but with poor tolerability. In both lung cancer cell lines A549 and H838, Liu et al. demonstrated a significant decrease in DNMT1 mRNA and protein expression levels by 5-Aza-CdR, which resulted in demethylation of RASSF1A in A549 but of RASSF1A, ASC, and APC in H838 [28]. This discrepancy of demethylation might implicate the different regulation mechanism in different cell lines. In our study, using another inhibitor 5-Aza-CR, the DNMT1 expression in A549 and H358 could be repressed to demethylate the promoter regions of RASSF1A and APC, resulting in re-expression of RASSF1A and APC. Additionally, the treatment of 5-Aza-CR also inhibited the cell proliferation and induced apoptosis in A549 and H358, which was similar to previous report [28]. Currently, the impacts of 5-Aza-CR on cell migration were unclear and we found that 5-Aza-CR significantly decreased the migration of A549 and H358.

As the novel agents of demethylation, the efficiency of siRNA-DNMTs should be compared to the DNMT inhibitor 5-Aza-CR. Suzuki et al. demonstrated that RNAi-Mediated knockdown of DNMT1 expression led to demethylation of tumor suppressor gene (RASSF1A, p16 ink4A, and CDH1) promoters in lung and breast cancer cell lines (NCI-H1299 and HCC1954), which restored the expression of RASSF1A and SEMA3B genes [29]. Another study *in vitro* also assessed the potential advantages of DNMT1-targeted inhibition for cancer therapy. They uncovered that siRNA-DNMT1, but not the siRNA-DNMT3B, induced the re-expression and reversed DNA methylation of five (CDKN2A, RASSF1A, HTLF, RUNX3, and AKAP12B) and showed to inhibit cell proliferation and induce cell death without DNA damage which could be induced by 5-Aza-CdR [18]. We here showed that, in lung cancer, siRNA-DNMT1 *in vitro* demethylated the RASSF1A and APC to restore its mRNA and protein expression, and induce apoptosis and the cell proliferation and migration were constricted *in vitro*. Importantly, the inhibitory effects of siRNA-DNMT1 on tumor progression were also observed *in vivo* and the side effects in mice treated with siRNA-DNMT1 were less than that treated with 5-Aza-CR, which indicated that the treatment of siRNA-DNMT1 could avoid the additional cytotoxicity unrelated to demethylation

In sum, we performed the comparison of efficiency and side effects between 5-Aza-CR and siRNA-DNMT1 *in vitro* and *in vivo* for lung cancer. We found siRNA-DNMT1, resemble to the 5-Aza-CR, could effectively demethylate the TSGs (e.g. RASSF1A and APC) to recover the tumor suppressive ability, but the treatment of siRNA-DNMT1 had less cytotoxicity than 5-Aza-CR which showed the potential superiority in lung cancer-therapy.

## MATERIALS AND METHODS

### Cell culture, tissue collection and reagents

The lung cancer cell line A549 and H358, and the normal lung epithelial cell BEAS-2B were purchased from the cell bank of the Chinese academy of sciences (Shanghai, China) and cultured in Dulbecco's Modified Eagle's medium (DMEM) supplemented with 10% FBS (Life Technologies, USA), ampicillin and streptomycin at 37 °C, 5% CO_2_ conditions. lung cancer tissues (n=20) and corresponding paracancerous tissues (n=20) were collected from department of Thoracic Surgery, the Affiliated Hospital of Guilin Medical University. All of the patients diagnosed with primary lung cancer were confirmed by hematoxylin and eosin staining by experienced pathologists. 5-Aza-CR was purchased from 5-Aza-CR (Sigma, St. Louis, MO). siRNA of DNMT1 (5’-GCCUCAUCGAGAAGAAUAUUU-3’) or control (5’-CAGAUGUUGCCAACAACAAGA-3’) was purchased from RiboBio (Guangzhou, china). Anti DNMT1, RASSF1A, APC and GAPDH antibodies were obtained from Cell Signaling Tech (Denver, MA).

### Methylation-specific PCR

DNA extracted from tissue samples was subjected to bisulfite modification to convert all unmethylated cytosines into uracils leaving methylated cytosines unmodified. The bisulfite modification was carried out by using the CpGenome™DNA modification kit (Chemicon International, Temecula, CA). Methylation-specific PCR (MSP) was performed using AmpliTaq® Gold with primers specific for methylated and unmethylated sequences of the genes. MSP primers for each gene were listed in Table 1.

**Table 1:** Primers for methylation-specific PCR

Gene	CPG status	Primers (5’-3’)	Product size (bp)
RASSF1A	Methylated	**Forward: TGTGATAGAAATTAAGGGGGTTTC**	223
		**Reverse: CAAATAAAAACCAAAAAATACCGAC**	
	Unmethylated	**Forward: GTGATAGAAATTAAGGGGGTTTTG**	224
		**Reverse: ACCAAATAAAAACCAAAAAATACCA**	
APC	Methylated	**Forward: GAGGGTATATTTTCGAGGGGTAC**	216
		**Reverse: TACACCAATACAACCACATATCGAT**	
	Unmethylated	**Forward: GAGGGTATATTTTTGAGGGGTATG**	216
		**Reverse: TACACCAATACAACCACATATCAAT**	

The treated DNA was used immediately or stored at−20°C until use. The bisulfite modified DNA was subjected to PCR. Positive control methylated DNA samples for each gene examined was used. Water blank was used as a negative control. PCR products were analyzed on 2.5% agarose gel and visualized under UV illumination.

### Cell transfection

Cells were seeded into 12 or 6-well plate and then A549 and H358 cells were transfected with siRNA-DNMT1 or negative control at a concentration of 100 nM by Lipofectamine 2000 (Invitrogen, USA) and cultured for indicated times.

### RNA isolation and qRT-PCR

Total RNA from tissues or A539 and H358 cells was extracted using Trizol reagent (Invitrogen) according to the standard RNA isolation protocol. Quantitative real-time RT-PCR (qRT-PCR) was performed, and the expression levels of DNMT1, RASSF1A, and APC were normalized to GAPDH for gene expression. The primers were listed in Table 2.

**Table 2:** Primers for qRT-PCR

Gene name	Primer
GAPDH-F	5’-ACACCCACTCCTCCACCTTT-3’
GAPDH-R	5’-TTACTCCTTGGAGGCCATGT-3’
DNMT1-F	5’-CCTCTATGGAAGGCTCGAGT-3’
DNMT1-R	5’-TCACCACACGGTGCTGCTCT-3’
RASSFA-F	5’-ACAGCAACCTCTTCATGAGCT-3’
RASSFA-R	5’-CAAGGAGGGTGGCTTCTTGCT-3’
APC-F	5’-TCCTGTCCCTGTATCAGAGACT-3’
APC-R	5’-ACTGTGTTTGCTTGAGCTGCT-3’

### Hoechst staining assay

A549 and H358 cell line treated with 5-Aza-CR transfected with siRNA-DNMT3b were cultured at 37°C for 24 or 48 hours, stained with 0.1 μg/ml Hoechst 33342 (Sigma, St Louis, MO, USA) was added to the culture medium; Fluorescence microscopy (OLYMPUS IX71; Olympus Corporation, Tokyo, Japan) with a filter for Hoechst 33342 (365 nm) was used to detected the changes of nuclear morphology.

### Flow cytometry assay

After the treatment of 5-Aza-CR or transfection with siRNA-DNMT3b, A549 and H358 cells were harvested and suspended with PBS, then cells were stained by for BrdU incorporation 8 hours after all experimental conditions with the BrdU Labeling and Detection Kit I (Roche). Cells were pre-labeled with BrdU were fixed with ethanol. Cells were then incubated with monoclonal antibodies against BrdU mixed with nucleases, followed by fluorescein-conjugated secondary antibodies according to the manufacturer's instructions and quantified by flow cytometry on a FACS Calibur instrument.

### Western blotting

After the treatment of 5-Aza-CR or transfection with siRNA-DNMT3b, A549 and H358 cells were harvested and total protein was isolated from the cell samples according to the manufacturer's protocol. Detailed procedures for immunoblotting are described elsewhere [18].

### Cell migration assays

The migration ability of A539 and H358 cells were measured by transwell chambers with an 8-μm pore size. For migration assays, 6 × 104 cells suspended in DMEM were seeded in the upper chamber and the lower chamber contained 500 μL DMEM/10 % FBS. The cells were incubated for 24 h and cells that did not migrate or invade were removed using a cotton swab. The cells were stained using crystal violet staining and counted under an inverted microscope. Four random views were selected to count the cells and the independent experiments were repeated three times.

### Tumor models

To investigate the efficiency of siRNA-DNMT1 in A549 model, 3×10^6^ A549 cells were subcutaneously injected in rear flank of nude mice (6 per group). 5-Aza-CR was administered by intraperitoneal injection (i.p.) at 500 nM/dose for five times, four days apart. The siRNA-DNMT1or negative control (10 nM per injection) were purchased from RiboBio (Guangzhou, China) and were delivered via intra-tumoral injection for six times, three days apart. Results are presented as the mean tumor size (mm^3^) and body weight (g) for every group at various time points until the termination of the experiment.

### Statistical analyses

The Statistical Package for Social Sciences version 16.0 (SPSS 16.0, SPSS Inc., Chicago, IL, USA) and the Prism statistical software package (Version 5.0, Graphpad Software Inc.) were used for the statistical analyses. Unpaired t-tests or Mann–Whitney U tests were used to compare the two groups, and multiple group comparisons were analyzed with one-way ANOVA. *P* < 0.05 was considered statistically significant. All experiments were performed at least three times.
